# Revealing key genes and molecular mechanisms associated with dietary restriction in ulcerative colitis

**DOI:** 10.3389/fmolb.2026.1786138

**Published:** 2026-03-25

**Authors:** Yingying Li, Min Xu, Wen Li, Hao Zhang, Qijin He, Shuyi Zhang

**Affiliations:** Department of Gastroenterology, Tianjin Union Medical Center, The First Affiliated Hospital of Nankai University, Tianjin, China

**Keywords:** ANGPTL4, CLDN1, CPT1a, dietary restriction, ulcerative colitis

## Abstract

**Introduction:**

Ulcerative colitis (UC) is characterized by chronic colonic mucosal inflammation, with its pathogenesis involving multidimensional interactions and limitations in clinical treatment. Dietary restriction (DR) is a commonly used approach for UC patients to alleviate symptoms, and exploring the role of DR-related genes in UC could provide new directions for the development of precision therapies.

**Methods:**

Bioinformatics analysis was performed on UC-related datasets (GSE75214, GSE73661) obtained from the GEO database. Candidate genes were acquired by intersecting differentially expressed genes (DEGs) with dietary restriction-related genes (DRRGs). Subsequently, key genes were identified via machine learning algorithms and ROC curve analysis. A deep neural network (DNN) model and a diagnostic nomogram were constructed. In addition, gene set enrichment analysis (GSEA), gene set variation analysis (GSVA), immune infiltration analysis, and single-cell RNA sequencing (scRNA-seq) analysis were conducted. Finally, the expression of key genes was validated through experiments.

**Results:**

CPT1A, ANGPTL4, and CLDN1 were identified as the key genes. The deep neural network (DNN) model achieved area under the curve (AUC) values of 0.914 and 0.933 in the two datasets, respectively; the diagnostic nomogram exhibited high predictive performance (AUC > 0.7), and decision curve analysis (DCA) revealed its potential clinical net benefit. Enrichment analyses demonstrated that the key genes were significantly enriched in dietary restriction (DR)-related pathways, including cytokine-receptor interaction, the IL2-STAT5 signaling pathway, and fatty acid metabolism. Thirty-two activated pathways and five inhibited pathways were detected in UC patients (e.g., the oxidative phosphorylation pathway was suppressed). Immune infiltration analysis identified 27 differentially infiltrating immune cell types. CLDN1 was localized to epithelial cells, ANGPTL4 to fibroblasts, and CPT1A to endothelial cells. Macrophages were identified as a signaling hub in UC, showing intensified crosstalk with stromal and vascular cells via pathways such as ACKR1. Experimental validation confirmed that ANGPTL4 and CLDN1 were highly expressed in UC, whereas CPT1A was lowly expressed, a pattern consistent with the expression trends observed in public database analyses.

**Discussion:**

These results indicated that CPT1A, ANGPTL4, and CLDN1 are involved in the pathological regulation of UC by DR through modulating the metabolism-immune-barrier axis, providing novel biomarkers and potential intervention targets for the clinical diagnosis and targeted therapy of UC.

## 1 Introduction

Inflammatory bowel disease (IBD), a chronic inflammatory condition, predominantly encompasses two major forms: ulcerative colitis and Crohn’s disease. The etiology of IBD is still unclear. The global prevalence of IBD has been steadily increasing in recent years (Hracs et al., 2025). Ulcerative colitis (UC) is a major subtype of IBD characterized by chronic persistent inflammation of the colonic mucosa, accompanied by crypt architectural distortion, mucosal ulceration, and abnormal immune cell infiltration. This chronic inflammation not only leads to recurrent diarrhea, mucopurulent bloody stool, abdominal pain, and other clinical symptoms, but also significantly increases the risk of UC-associated colorectal cancer (Gros and Kaplan, 2023). Despite recent advancements in UC pathogenesis research, which has been found to involve multi-dimensional interactions among genetic susceptibility, intestinal microbiota dysbiosis, mucosal immune imbalance, and intestinal mucosal barrier injury, current clinical treatment still faces challenges (Kobayashi et al., 2020). Conventional therapies, including 5-aminosalicylic acid (5-ASA), corticosteroids, and immunosuppressive agents, primarily focus on symptom control and remission induction, with limited efficacy in modifying disease progression or reducing cancer risk (Lipka et al., 2013). Although biologics have significantly improved clinical outcomes, they exhibit considerable interindividual variability in treatment response, and their long-term safety profiles remain to be fully elucidated (Kucharzik et al., 2020). Accumulating evidence suggests that UC pathogenesis involves complex interactions between host genetics, immune dysregulation, environmental factors, and intestinal barrier dysfunction.3 Diet (one of the environmental factors) is closely related to the occurrence and development of UC. Mounting evidence suggests that dietary modifications can trigger intestinal dysbiosis and aberrant immune responses in UC patients (Jing et al., 2025; Ye et al., 2025). Therefore, in-depth elucidation of the molecular networks underlying the pathogenesis and progression of UC, particularly the identification of key disease-modifying genes and therapeutic targets, represents a critical research priority to overcome current therapeutic limitations and advance precision medicine in this field.

Dietary restriction (DR), encompassing caloric restriction and other restrictive dietary interventions, refers to the practice of reducing overall nutrient intake while maintaining adequate provision of essential amino acids, vitamins, and micronutrients to prevent malnutrition (Oswald et al., 2025). Research has established dietary restriction (DR) as the most robust non-pharmacological strategy for cancer chemoprevention in rodents and non-human primates (Oswald et al., 2025). Beyond its anti-tumorigenic properties, DR also represents the most effective non-genetic intervention measure to prolong the lifespan of rodents and rhesus monkeys (Di Francesco et al., 2024; Mattison et al., 2017), and mounting evidence indicates its capacity to modulate the pathogenesis and progression of multiple age-related disorders across rodents and humans. In the context of human health, DR exerts its protective effects by reducing growth factors and anabolic hormones, suppressing inflammatory cytokines, and mitigating oxidative stress and free radical-mediated DNA damage (Green et al., 2022; McAuley, 2022; Aminzadeh-Gohari et al., 2024).

Diet, as a modifiable environmental factor, has been implicated in the initiation and progression of UC. Accumulating clinical and epidemiological evidence has demonstrated that adherence to certain dietary patterns confers beneficial effects on UC management (Erol Doğan et al., 2024; Kedia et al., 2024). However, the specific molecular mechanisms underlying how these dietary factors and dietary restriction strategies regulate UC pathogenesis remain poorly elucidated, representing a critical knowledge gap in the field (Campmans-Kuijpers and Dijkstra, 2021; Jiang et al., 2021).

To address this gap, this study aims to evaluate the expression patterns and functional roles of DR-related genes (DRRGs) in UC through bioinformatics analysis. We hypothesize that DRRGs exhibit a specific expression profile in the mucosal tissues of UC patients; these genes may participate in the pathological processes of UC by regulating metabolism, immune response, or intestinal barrier function, and thus hold potential as novel biomarkers or therapeutic targets for UC.

## 2 Materials and methods

### 2.1 Source of data

The Gene Expression Omnibus (GEO) (http://www.ncbi.nlm.nih.gov/geo/) database was utilized to obtain the UC-related datasets (GSE75214 and GSE73661) both based on the microarray platform GPL6244 (Affymetrix Human Gene 1.0 ST Array). Normalized Series Matrix files (background-corrected and quantile-normalized) were used, with expression values further log2-transformed. As a training set, the GSE75214 dataset comprised 74 colon mucosal tissue samples from UC patients and 11 control samples (Vancamelbeke et al., 2017). The GSE73661 dataset was utilized as a validation set, containing 67 colon mucosal tissue samples from UC patients and 12 control samples (Arijs et al., 2018). Probe IDs were mapped to gene symbols; for multiple probes mapping to one gene, the probe with the maximum average expression was retained. No additional low-expression filtering was applied to ensure analysis integrity. Additionally, the single-cell RNA sequencing dataset GSE231993, including colon tissue samples from four ulcerative colitis patients and four healthy controls, was obtained from the GEO database to explore cell-type-specific gene expression and immune cell heterogeneity in UC tissues.

Based on prior research (Song et al., 2024), 276 dietary restriction-related genes (DRRGs) were retrieved (Supplementary Table S1). Integrated from multiple resources (including GenDR), this gene set has broad biological representativeness, validated dietary restriction associations, and tight links to core ulcerative colitis pathways (notably metabolism/immunity). To further validate the initial 276 dietary restriction-related genes (DRRGs) (Song et al., 2024), we supplemented analysis with 174 DR genes downloaded from HAGR’s Supplementary Table S2 (https://hagr.ageing-map.org/diet/TableS2.xls). Intersection of the two gene sets yielded 130 overlapping genes, which included our three key genes (CPT1A, ANGPTL4, CLDN1) The Venn diagram illustrating this gene overlap and the detailed list of intersecting genes are provided in Supplementary Figure S1 and Supplementary Table S21. This confirms the rationality of selecting the original DRRGs.

### 2.2 Identification of transcriptional expression differences

Transcriptional profiling analysis comparing UC patients and control subjects was conducted using the “limma” computational package (v 3.54.0) (Ritchie et al., 2015) within the GSE75214 dataset, with the objective of identifying DEGs. Gene screening was performed employing filtering thresholds of P.adj <0.05 combined with |log2Fold Change (FC)| >1. Visualization of DEGs expression patterns across GSE75214 was achieved through volcano plot construction and heatmap generation, implemented using the “ggplot2″ package (v 3.5.1) (Gustavsson et al., 2022) and “ComplexHeatmap” package (v 2.21.1) (Gu and Hübschmann, 2022), respectively. The top 10 upregulated and downregulated DEGs, ranked based on their P.adj values, were prominently featured in both volcano plot and heatmap visualizations.

### 2.3 Target gene derivation, functional characterization, and chromosomal mapping

Intersection analysis between DEGs and DRRGs was executed using the “VennDiagram” package (v 1.7.3) (Chen and Boutros, 2011) to derive target genes. Subsequently, chromosomal distribution patterns of target genes were investigated through genomic localization analysis. The chromosomal mapping analysis was performed utilizing the “RCircos” package (v 1.2.2) (Zhang et al., 2013). To investigate associated biological mechanisms and regulatory pathways, Gene Ontology (GO) and Kyoto Encyclopedia of Genes and Genomes (KEGG) enrichment analyzes (P < 0.05) were conducted on target genes employing the “clusterProfiler” package (v 4.8.3) (Wu et al., 2021). For visualization objectives, the top 3 statistically significant terms within each GO analysis section were selected. Specifically, GO biological functions encompass cellular component (CC), molecular function (MF), and biological process (BP) categories.

### 2.4 Acquisition of characteristic genes

Target gene candidates were submitted to the Search Tool for the Retrieval of Interacting Genes/Proteins (STRING) platform (v 11.5, http://www.string-db.org/), configuring the organism parameter as ‘*Homo sapiens*’ with a minimum interaction confidence score of ≥0.15 to establish the protein-protein interaction (PPI) regulatory network. Gene prioritization was performed utilizing multiple centrality measurement approaches within the Cytohubba analytical plugin, including Betweenness centrality (BC), Edge Percolated Component (EPC), Maximal Clique Centrality (MCC), and Radiality (RC) computational algorithms. The highest-ranking 10 genes identified by each centrality algorithm were subjected to intersection analysis using the “VennDiagram” computational package (v 1.7.3), with convergent genes designated as candidate characteristic molecular markers.

Computational machine-learning methodologies were implemented on candidate characteristic molecular markers within the GSE75214 dataset to discriminate characteristic genes. Specifically, the least absolute shrinkage and selection operator (LASSO) penalized regression modeling was conducted utilizing the “glmnet” statistical package (v 4.1.8) (https://www.jstatsoft.org/v33/i01) (optimal predictive model selection was achieved at minimal lambda parameter value), whereas support vector machine recursive feature elimination (SVM-RFE) algorithmic analysis was performed through the “caret” machine learning framework (v 6.0.94) (Kuhn, 2008) (implementing minimal classification error optimization principle). Critically, 10-fold cross-validation methodology was employed during LASSO regression analysis to ensure model robustness. Subsequently, gene candidates derived from both algorithmic approaches were intersected to determine final characteristic genes, with visualization of results accomplished using the “VennDiagram” package (v 1.7.3).

### 2.5 Identification of key genes

To evaluate the discriminative capability of characteristic genes for differentiating UC specimens from healthy control specimens, diagnostic performance curves based on receiver operating characteristic (ROC) methodology were constructed, and the area under the receiver operating characteristic curve (AUC) metrics were computed for characteristic genes across the discovery cohort GSE75214 and verification cohort GSE73661. Genes demonstrating AUC values >0.7 were designated as potential biomarker genes (AUC >0.7 suggesting robust diagnostic accuracy). Non-parametric Wilcoxon rank-sum statistical tests were conducted to assess transcriptional variations of potential biomarker genes among UC patients and healthy controls within both independent cohorts. Genes exhibiting statistically significant between-group variations and concordant transcriptional patterns across both discovery and verification datasets were defined as critical biomarker genes (P < 0.05).

To evaluate the clinical predictive value of key genes, a diagnostic nomogram was constructed using the “rms” R package (v 6.5.0). The predictive accuracy of the model was assessed via calibration curves and receiver operating characteristic (ROC) analysis with 10-fold cross-validation (using the “pROC” package, v 1.18.0). An area under the curve (AUC) > 0.7 was considered to indicate high diagnostic value. Furthermore, decision curve analysis (DCA) was performed using the “rmda” package (v 1.6) to determine the clinical net benefit and decision-making utility of the nomogram.

### 2.6 Deep neural network (DNN)

Based on the median expression value of each key gene in the GSE75214 set, samples were assigned a value of ‘0′or ‘1′according to their expression levels. Subsequently, subjects were partitioned into high and low expression categories according to their expression levels. The matrix was input into the “neuralnet” package (v 1.44.2) (Li et al., 2023) to construct a three-layer feed-forward DNN (comprising 3 input, 5 hidden, and 1 output neurons). The model was trained using the rprop + algorithm with a logistic activation function and a termination threshold of 0.15. This process determined the weights of the key genes (the thicker the line, the greater the weight). The model trained on GSE75214 was then directly applied to GSE73661 without retraining. After that, a calculated AUC greater than 0.7 indicated that the DNN had good predictive ability for UC (GSE75214 and GSE73661) (AUC >0.7 indicating good diagnostic efficacy).

### 2.7 Gene set enrichment analysis (GSEA) and gene set variation analysis (GSVA)

Gene Set Enrichment Analysis (GSEA) methodology was employed to elucidate the molecular mechanisms and biological processes associated with pivotal genes contributing to UC pathogenesis. Spearman rank correlation analysis was conducted utilizing the “psych” computational package (v 2.4.3) (Zein and Akhtar, 2025) to determine correlation relationships between individual pivotal genes and the entire transcriptome across all specimens within the GSE75214 cohort. Following correlation computation, genes were systematically arranged in descending order according to their correlation coefficient values, generating ranked gene lists corresponding to each pivotal gene. Subsequently, GSEA enrichment analysis was executed utilizing the curated reference gene collection “c2.cp.kegg.v7.5.1.symbols.gmt” obtained from the Molecular Signatures Database (MSigDB) repository (https://www.gsea-msigdb.org/gsea/msigdb) (statistical significance threshold: P < 0.05, |Normalized Enrichment Score (NES)| > 1). Visualization was performed for the most statistically significant 5 regulatory pathways (arranged by ascending P values) derived from pivotal gene enrichment outcomes. To investigate molecular pathway alterations between UC patients and healthy controls within the GSE75214 dataset, the hallmark gene collection “h.all.v2024.1.Hs.symbols.gmt” was retrieved from the MSigDB database repository. Gene Set Variation Analysis (GSVA) enrichment assessment was conducted on the complete transcriptome of UC patients and control subjects using the “GSVA” analytical package (v 1.50.0) (Hänzelmann et al., 2013), subsequently followed by comparative analysis of GSVA enrichment scores between UC cohort and control cohort utilizing the “limma” statistical framework (v 3.54.0) (significance criteria: P < 0.05; |t-statistic| >2).

### 2.8 Immune infiltration analysis

Immune infiltration analysis was applied to probe the differences in the immune microenvironment between the UC group and the control group in the GSE75214 set. The infiltration abundance of 28 immune cells (Jiang et al., 2022) was computed using the ssgsea algorithm. The infiltration abundance of these immune cells between the UC and control groups was demonstrated by a heat map (P < 0.05). The differences in these immune cells between groups were examined using the Wilcoxon test to obtain differential immune cells (DICs) (P < 0.05). Right after that, the “psych” package (v 2.4.3) was utilized to perform a Spearman correlation analysis, aiming to explore the potential associations both among DICs and between each key gene and DICs (|correlation (cor)| >0.3, P < 0.05).

### 2.9 Friends analysis

To investigate the functional similarity among key genes, functional similarity scores were calculated for key genes using the “GOSemSim” package (v 2.26.1) (Yang et al., 2023) based on UC samples from the GSE75214 dataset.

### 2.10 Molecular regulatory network and drug prediction

Common microRNAs (miRNAs) of key genes were predicted through 3 databases: microRNA walkthrough database (miRWalk) (v 3.0, http://mirwalk.umm.uni-heidelberg.de/), target scanning for microRNA targets (TargetScan) (v 8.0, https://www.targetscan.org/vert_80/) and SRNA-Target association repository (starbase) (v 3.0, https://rnasysu.com/encori/). Transcription factors (TFs) of key genes were predicted using the joint article supporting publication association for regulatory factors in eukaryotes (JASPAR) database (https://jaspar.elixir.no/). The predicted common miRNAs and TFs were imported into Cytoscape software (v 3.9.1) (Smoot et al., 2011) for visualization. To predict potential drugs for treating UC, the DSigDB database (https://dsigdb.tanlab.org/DSigDBv1.0) was used for retrieval.

### 2.11 Single-cell RNA-sequencing (scRNA-seq) analysis

ScRNA-seq data from GSE231993 (4 UC, 4 controls) were analyzed using Seurat (v4.3.0). Cells with 200–6,000 genes, >20,000 total mRNA, or >10% mitochondrial content were excluded. After normalization, 2,000 highly variable genes were identified for PCA. The top 30 PCs were used for unsupervised clustering (resolution = 0.2) and UMAP visualization. Cell types were annotated based on previous literature and CellMarker 2.0 (Mors et al., 2019) (marker genes were listed in Supplementary Table S22). Cell type abundance was compared using ggplot2, and intercellular communication (ligand-receptor pairs) was evaluated using CellChat (P < 0.05, Log2mean ≥0.1).

### 2.12 Quantitative reverse transcription polymerase chain reaction (RT-qPCR) validation

To validate and corroborate findings derived from public repository analysis, UC tissue specimens (n = 5) were obtained from 5 patients with moderately active ulcerative colitis who were hospitalized at The First Affiliated Hospital of Nankai University. Healthy control specimens were obtained from 5 normal individuals. RT-qPCR validation experiments were conducted utilizing these clinical specimens. Ethical approval for this investigation was obtained from the hospital’s Institutional Ethics Review Board (Authorization No.: C10, 2022). The detailed clinical characteristics of the enrolled participants are summarized in Supplementary Table S23. Prior to experimental procedures, written informed consent was secured from all study participants through signed consent documentation. According to the manufacturer’s specifications, total ribonucleic acid (RNA) extraction was performed from 10 specimens using TRIzol reagent (Vazyme, China). Complementary DNA (cDNA) synthesis was accomplished using the HP All-in-one qRT Master Mix II RT203-Ver.1 reverse transcription system (Kunming Yungen Biotechnology Co., Ltd). Following reverse transcription, quantitative polymerase chain reaction (qPCR) amplification experiments were executed. To ensure accurate assessment of transcriptional expression levels, data normalization was performed using the reference housekeeping gene glyceraldehyde-3-phosphate dehydrogenase (GAPDH). Relative transcriptional expression quantification was calculated employing the comparative 2^−^ΔΔCt analytical method, consistent with established protocols described in previous literature (P < 0.05) (Shang et al., 2022).

### 2.13 Statistical methodology

Data processing and analysis were performed utilizing R statistical computing environment (v 4.2.2). Non-parametric Wilcoxon rank-sum tests and parametric Student's t-tests were employed to evaluate statistical differences among comparative groups. Statistical significance was established at a P value threshold less than 0.05.

## 3 Results

### 3.1 Acquisition of candidate genes

**FIGURE 1:**
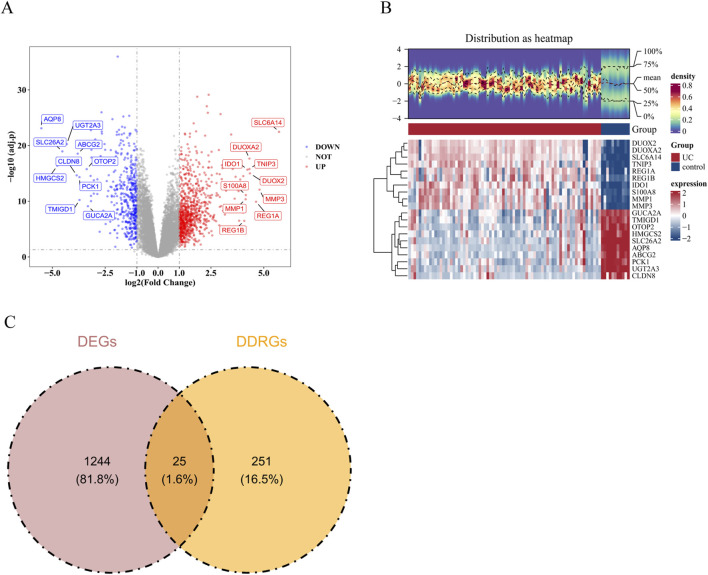
Identification of differentially expressed genes (DEGs). **(A)** Volcano plot of DEGs. **(B)** Heatmap of DEGs. **(C)** Identification of candidate genes. Complete names and details of these 25 candidate genes are in Supplementary Table S3.

Altogether, 1269 DEGs were identified between the UC and control groups in the GSE75214 dataset. Among these DEGs, 819 genes exhibited significant upregulation in the UC compared with control groups, while 450 genes showed significant downregulation (P.adj <0.05, |log2FC| >1) (Figures 1A,B; Supplementary Table S2). Then, these DEGs were intersected with DRRGs, resulting in the identification of 25 candidate genes (Figure 1C; Supplementary Table S3).

### 3.2 Functional analysis and chromosomal localization

**FIGURE 2:**
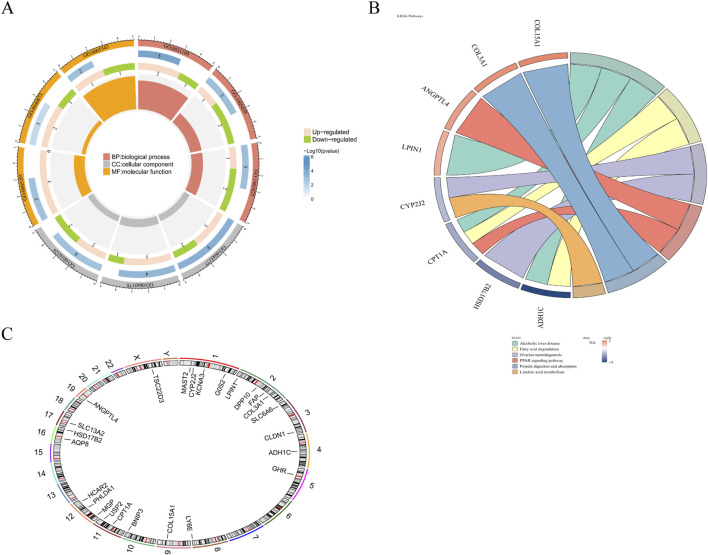
Functional analysis and chromosomal localization. **(A)** GO enrichment analysis of candidate genes. **(B)** KEGG enrichment analysis of candidate genes. **(C)** Chromosomal localization of candidate genes.

To elucidate the biological roles and molecular pathways linked to the candidate genes, we conducted a functional enrichment analysis. The findings indicated that these candidate genes showed remarkable enrichment in 274 GO entries, which included 196 BPs, 22 CCs, and 56 MFs (P < 0.05) (Supplementary Table S4). These entries included apical plasma membrane, sodium ion transmembrane transporter activity, inflammatory cell apoptotic process, and so on (Figure 2A). Furthermore, these candidate genes demonstrated significant enrichment in 6 KEGG signaling pathways, including but not limited to fatty acid degradation and protein digestion and absorption pathway (P < 0.05) (Figure 2B; Supplementary Table S5). In addition, Chromosomal localization analysis showed that the genes MAST2, CYP2J2, G0S2, and KCNA3 were all localized on chromosome 1, while the genes LPIN1, DPP10, FAP, and COL3A1 were all localized on chromosome 2 (Figure 2C).

### 3.3 Identification of key genes

**FIGURE 3:**
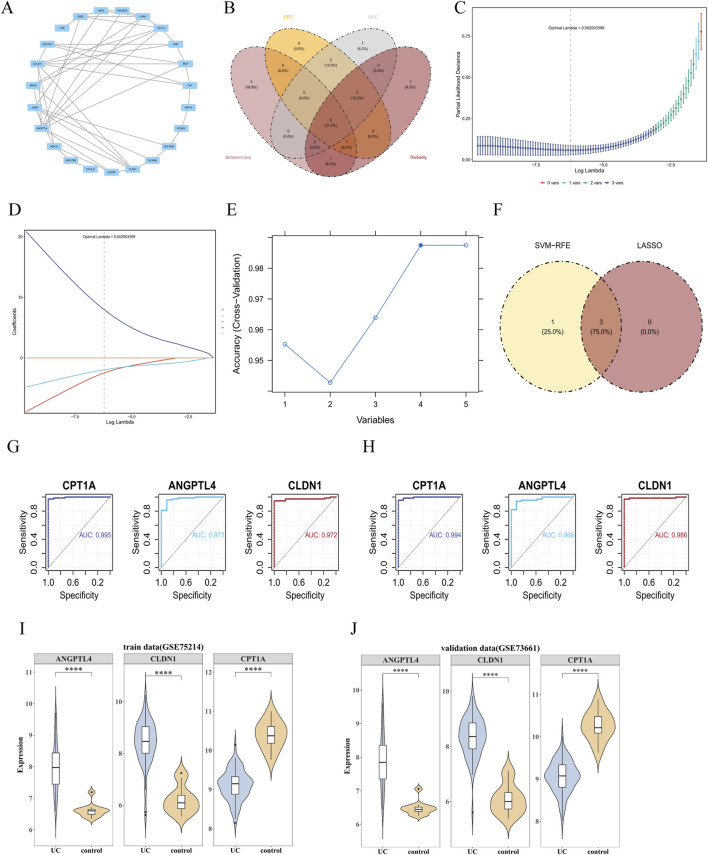
Identification of key genes. **(A)** PPI network of candidate genes. **(B)** Intersection of the top 10 genes from four algorithms. **(C)** Schematic diagram of the model error from the LASSO regression screening results. **(D)** Coefficient profile plot from the LASSO regression. **(E)** Results of the SVM-RFE analysis. **(F)** Identification of key genes. **(G)** ROC analysis of the feature genes in the training set GSE75214. **(H)** ROC analysis of the feature genes in the validation set GSE73661. **(I)** Expression of candidate biomarkers in UC patient samples and control samples from the training set GSE75214. **(J)** Gene expression of the candidate biomarkers in UC samples and control samples from the validation set GSE73661.

PPI analysis showed that 23 candidate genes exhibited protein-protein interactions (Figure 3A). Through the intersection of the top 10 genes from the BC (Supplementary Table S6), EPC (Supplementary Table S7), MCC (Supplementary Table S8), and RC (Supplementary Table S9) algorithms, 5 candidate characteristic genes (COL3A1, ANGPTL4, CLDN1, G0S2, and CPT1A) were identified (Figure 3B). LASSO analysis showed that when lambda = 0.002003399, the regression coefficients of 3 genes (ANGPTL4, CLDN1, and CPT1A) were not penalized to zero (Figures 3C,D). In the SVM-RFE algorithm, the model achieved the highest prediction accuracy when the number of genes was 4, with the corresponding genes being CPT1A, COL3A1, ANGPTL4, and CLDN1 (Figure 3E). The intersection of these 2 sets yielded 3 characteristic genes: ANGPTL4, CLDN1, and CPT1A (Figure 3F).

ROC analysis in GSE75214 and GSE73661 showed that the AUC values of genes CPT1A, ANGPTL4, and CLDN1 were all greater than 0.9; thus, these genes were considered as candidate key genes (Figures 3G,H). Further expression analysis in GSE75214 and GSE73661 indicated that CPT1A, ANGPTL4, and CLDN1 exhibited intergroup differences in both datasets with consistent expression trends, leading to their final identification as key genes (P < 0.05) (Figures 3I,J).

### 3.4 Construction of a DNN for UC prediction

**FIGURE 4:**
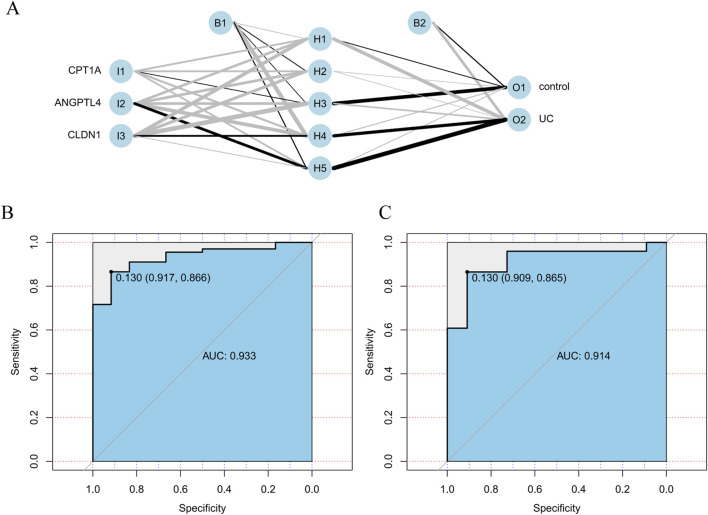
Construction of a DNN for UC prediction. **(A)** Neural network model constructed in the training set. **(B,C)** ROC analysis.

In the construction of the DNN based on the key genes (CPT1A, ANGPTL4, and CLDN1), ANGPTL4 had a stronger tendency to be associated with the middle layers ANGPTL4 had a higher weight in the UC group (Figure 4A)

In the GSE75214 and GSE73661 datasets, the AUC values of the ROC curves for the DNN, with AUC >0.7, were 0.914 and 0.933, respectively (Figures 4B,C). This demonstrated that the DNN exhibited good predictive ability for distinguishing between UC and control samples (AUC >0.9, indicating good diagnostic efficacy).

### 3.5 Construction and clinical evaluation of the diagnostic nomogram

**FIGURE 5:**
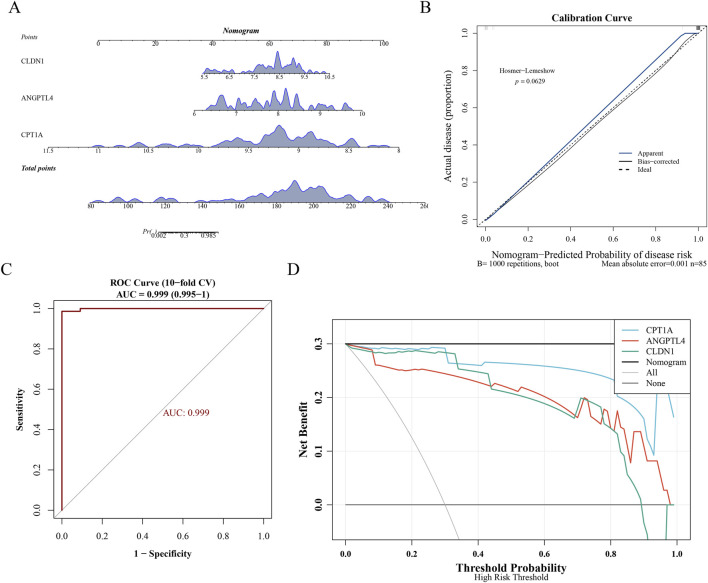
Diagnostic nomogram and its clinical performance. **(A)** Nomogram model: A point-based scoring system where red lines indicate the risk calculation for a representative sample based on key gene expression. **(B)** Calibration curve: Assessment of predictive accuracy (Blue: actual; Black: bias-corrected via 100-repetition bootstrapping). **(C)** ROC curve: Evaluation of diagnostic performance (AUC = 0.999). **(D)** DCA curve: Clinical utility analysis showing net benefit across high-risk thresholds.

To further facilitate the clinical application of the key genes (CPT1A, ANGPTL4, and CLDN1), a diagnostic nomogram was developed to predict the risk of UC (Figure 5A). In this model, each gene’s expression level contributes to a total score that corresponds to a specific disease probability. The calibration curve demonstrated high consistency between the predicted and actual probabilities (Figure 5B). Moreover, the nomogram achieved an outstanding diagnostic performance with an AUC of 0.999 (Figure 5C). Decision curve analysis (DCA) further confirmed that the model provides significant clinical net benefit across a wide range of threshold probabilities (Figure 5D), highlighting its potential utility in personalized UC diagnosis.

### 3.6 GSEA and GSVA

**FIGURE 6:**
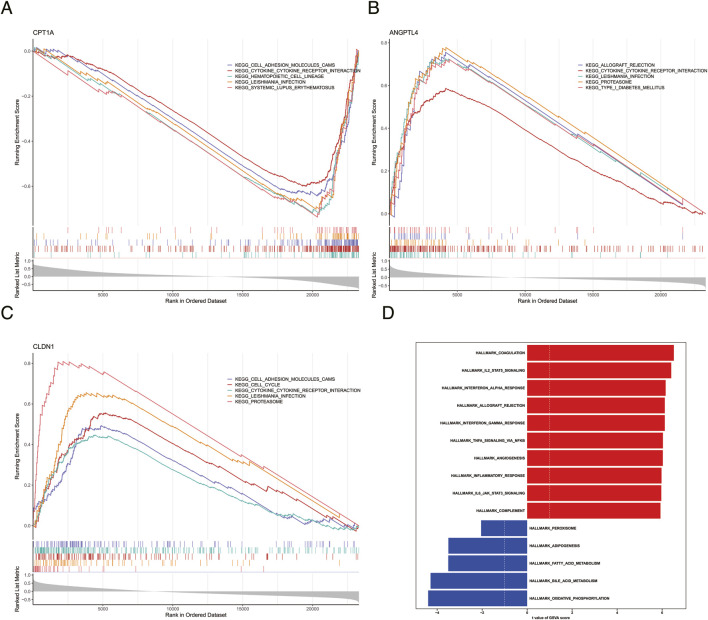
GSEA and GSVA. **(A)** GSEA enrichment analysis results for the biomarker CPT1A. **(B)** GSEA enrichment analysis results for the biomarker ANGPTL4. **(C)** GSEA enrichment analysis results for the biomarker CLDN1. **(D)** Schematic diagram of the GSVA analysis results.

GSEA enrichment analysis showed that CPT1A, ANGPTL4, and CLDN1 were enriched in 99 (Figure 6A; Supplementary Table S10), 109 (Figure 6B; Supplementary Table S11), and 93 (Figure 6C; Supplementary Table S12) pathways, respectively. Including pathways such as pathways in cytokine-receptor interaction and leishmania infection (P < 0.05, |NES| >1). Additionally, GSVA enrichment analysis exhibited that there were 37 significantly differentially expressed pathways between the UC group and control group, with 32 pathways upregulated and 5 pathways downregulated (P < 0.05; |t| >2) (Supplementary Table S13). Pathways such as IL2 STAT5 signaling and interferon alpha response were activated in the UC group, while pathways like oxidative phosphorylation and bile acid metabolism were inhibited in the UC group (Figure 6D).

### 3.7 Immune cell infiltration and friends analysis

**FIGURE 7:**
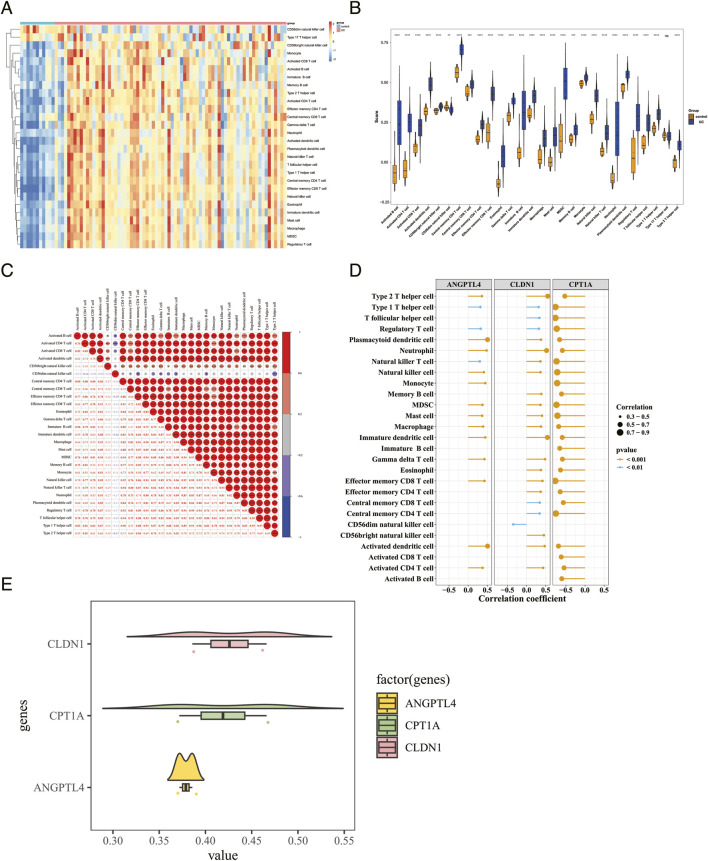
Immune cell infiltration and Friends analysis. **(A)** Infiltration levels of 28 immune cell types in the UC group and the control group. **(B)** Screening of differentially abundant immune cells. **(C)** Spearman correlation analysis among the differentially abundant immune cells. **(D)** Correlation analysis between the biomarkers and the differentially abundant immune cells. **(E)** Functional similarity analysis among the biomarkers.

Within the GSE75214 cohort, the percentage of neutrophilic granulocytes in UC patients was markedly elevated compared to healthy controls (Figure 7A). A comprehensive total of 27 differentially infiltrating immune cells (DICs) were systematically evaluated (statistical significance: P < 0.05) (Figure 7B). Cellular infiltration patterns of all immune cell populations, with the exception of T helper 17 lymphocytes, exhibited significant variations between comparative groups. Analysis revealed that myeloid-derived suppressor cells (MDSCs) and activated dendritic cell populations demonstrated robust positive association (correlation coefficient = 0.94, P < 0.05), whereas T helper type 2 lymphocytes and CD56dim natural killer cell subsets displayed moderate inverse correlation (correlation coefficient = −0.47, P < 0.001) (Figure 7C). Furthermore, the majority of DICs exhibited positive associations with angiopoietin-like protein 4 (ANGPTL4) and claudin-1 (CLDN1), while demonstrating inverse correlations with carnitine palmitoyltransferase 1A (CPT1A) (|correlation coefficient| >0.3, P < 0.05) (Figure 7D; Supplementary Table S14). Moreover, functional relationship network analysis revealed that the CLDN1 gene exhibited the highest degree of molecular resemblance with both ANGPTL4 and CPT1A genes (Figure 7E).

### 3.8 Molecular regulatory network and drug prediction

**FIGURE 8:**
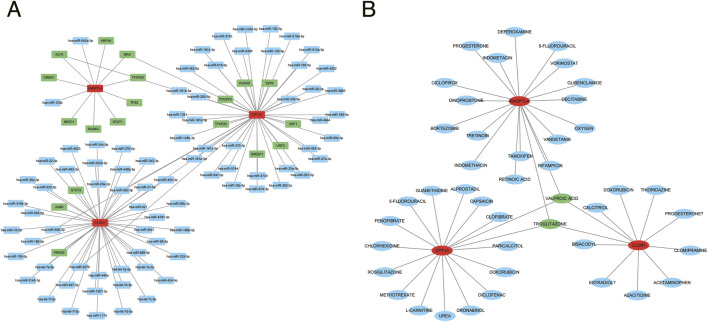
Molecular regulatory network and drug prediction. **(A)** TF-miRNA-mRNA regulatory network. **(B)** Key gene-drug interaction network.

miRNA prediction for key genes showed that ANGPTL4 had 2 common miRNAs across miRWalk, TargetScan, and starbase databases; CLDN1 had 43 common miRNAs in the 3 databases; and CPT1A had 39 common miRNAs (Supplementary Table S15). TF prediction indicated that ANGPTL4, CLDN1, and CPT1A were predicted to have 9, 3, and 10 TFs, respectively, in the JASPAR database (Supplementary Table S16). As shown in Figure 8A, it is a TF-miRNA-mRNA regulatory network, such as POU2F2-CPT1A-hsa-miR-483–5p.

Drug prediction showed that ANGPTL4 was associated with 78 drugs (including 19 Food and Drug Administration (FDA)-approved drugs) (Supplementary Table S17), CLDN1 with 59 drugs (including 11 FDA-approved drugs) (Supplementary Table S18), and CPT1A with 68 drugs (including 17 FDA-approved drugs) (Supplementary Table S19). Figure 8B showed the key gene-drug network of FDA-approved drugs, in which troglitazone and valproic acid could target all 3 key genes simultaneously.

### 3.9 Single-cell expression landscape and intercellular communication

**FIGURE 9:**
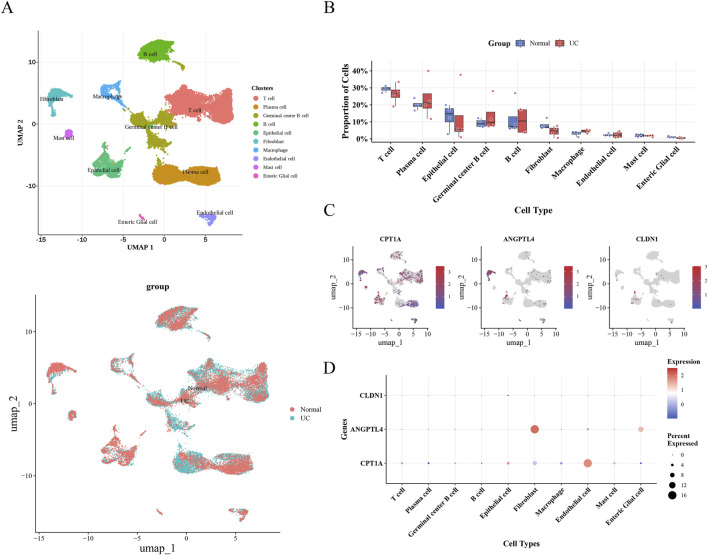
Single-cell expression landscape and distribution of key biomarkers in UC. **(A)** UMAP visualization of 26,305 single cells from the GSE231993 dataset, annotated into 10 distinct cell types (left) and colored by group (Normal vs. UC, right). **(B)** Box plot comparing the proportion of identified cell types between the Normal and UC groups. **(C)** Feature plots showing the expression distribution of three key biomarkers (CPT1A, ANGPTL4, and CLDN1) across the single-cell UMAP landscape. **(D)** Dot plot illustrating the expression levels and percentage of cells expressing CPT1A, ANGPTL4, and CLDN1 across the 10 annotated cell types. The color scale represents the average expression level, and the dot size represents the percentage of cells expressing the gene.

To validate the identified biomarkers at single-cell resolution, we analyzed the GSE231993 dataset. After rigorous quality control (QC), 26,305 high-quality cells were retained (Supplementary Figure S2A). Feature selection and dimensionality reduction through PCA and UMAP categorized cells into 10 distinct clusters (Supplementary Figure S2B). Based on canonical marker genes, these clusters were annotated as T cells, Plasma cells, Germinal center B cells, B cells, Epithelial cells, Fibroblasts, Macrophages, Endothelial cells, Mast cells, and Enteric Glial Cells (Figure 9A; Supplementary Figure S2C).

Cellular composition analysis revealed significant heterogeneity between the UC and control groups. Notably, the abundance of Macrophages was markedly higher in the UC group compared to healthy controls, suggesting their pivotal role in the inflammatory microenvironment (Figure 9B). Consistent with this, we designated macrophages as a key cell population for further analysis.

We then projected the expression of the three biomarkers onto the single-cell landscape. CPT1A was widely expressed across multiple cell types, with relatively higher levels in endothelial cells. ANGPTL4 was predominantly enriched in the fibroblast cluster, while CLDN1 exhibited low overall expression in this specific dataset (Figures 9C,D).

**FIGURE 10:**
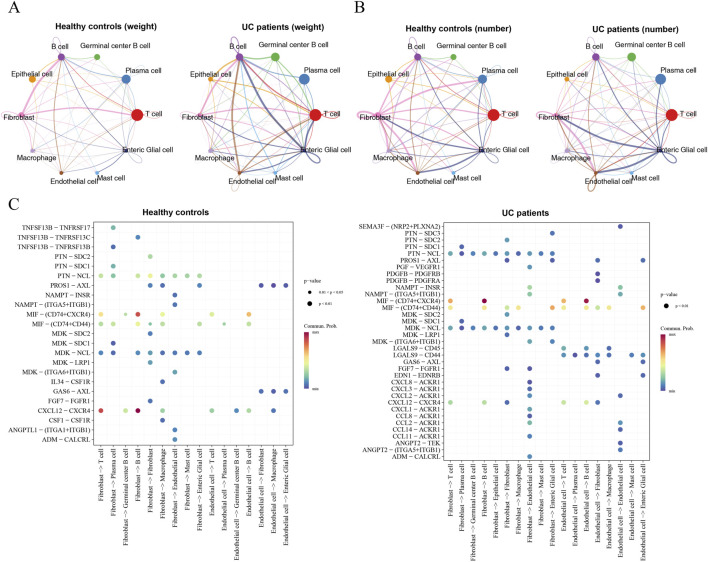
Intercellular communication landscape in UC microenvironment via CellChat analysis. **(A)** Circle plots comparing the total interaction strength (weight) between various cell types in Healthy controls (left) and UC patients (right). Thicker lines indicate stronger interaction intensity. **(B)** Circle plots illustrating the total number of interactions among cell clusters in Healthy controls (left) and UC patients (right). **(C)** Bubble plots showing selected ligand-receptor pairs and their communication probabilities. The x-axis represents the direction of communication, and the y-axis represents specific signaling pathways. The color scale indicates the communication probability, and the dot size represents the p-value.

Intercellular communication analysis via CellChat demonstrated that the communication network was significantly altered in UC patients. In the UC group, macrophages exhibited strong interaction counts with fibroblasts and endothelial cells, and high interaction strength with B cells (Figures 10A,B). Specifically, UC-derived fibroblasts showed increased communication with macrophages compared to the control group. Furthermore, communication signaling pathways such as CXCL12 were attenuated between T/B cells in UC, while endothelial cell interactions via the ACKR1 receptor were significantly enhanced (Figure 10C). These findings suggest that the identified biomarkers, particularly ANGPTL4 and CPT1A, may participate in UC pathogenesis by modulating the complex crosstalk between macrophages, fibroblasts, and the vascular endothelium.

### 3.10 Quantification of key gene expression

**FIGURE 11:**
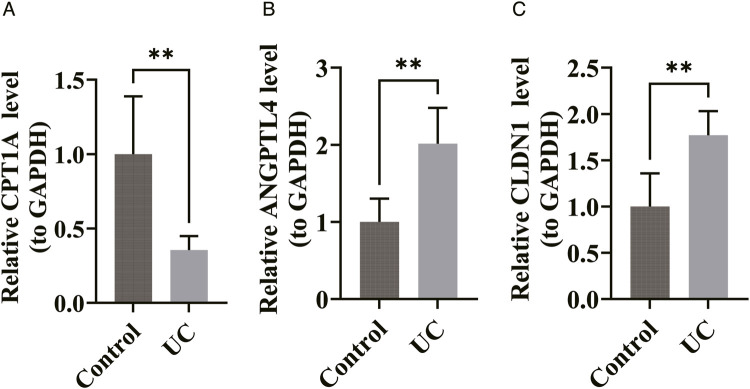
Quantification of key gene expression. **(A)** The quantification results of CPT1A. **(B)** The quantification results of ANGPTL4. **(C)** The quantification results of CLDN1. **p < 0.01.

Transcriptional expression patterns of pivotal genes within patients with moderately active ulcerative colitis and healthy control subjects were assessed through quantitative reverse transcription polymerase chain reaction (RT-qPCR) methodology (oligonucleotide primer sequences detailed in: Supplementary Table S20). Remarkably, relative to the healthy control cohort (P < 0.05), angiopoietin-like protein 4 (ANGPTL4) and claudin-1 (CLDN1) transcriptional levels were markedly elevated within the UC patient cohort, while carnitine palmitoyltransferase 1A (CPT1A) transcriptional levels were substantially reduced, with significant statistical variations detected between comparative groups (Figure 11). The quantification results of ANGPTL4, CLDN1, and CPT1A expression were consistent with the expression trends from the public database analysis.

## 4 Discussion

IBThe global incidence of IBD has been on a steady rise, with DR emerging as a significant environmental factor. As a crucial environmental factor, yet, the regulatory mechanisms of its associated genes in UC remain unclear (Ng et al., 2017; Andersen et al., 2018). In the present study, through screening the intersection of DR-related genes (DRRGs) and UC differentially expressed genes (DEGs), has for the first time identified CPT1A, ANGPTL4, and CLDN1 as key DR-associated biomarkers in ulcerative colitis. The entire process strictly adhered to the core research focus on “UC and DR-related genes”, ensuring rigorous continuity of the screening workflow. The core innovation of this study lies in confirming that the expression alterations of these genes are closely associated with DR. Through pathway enrichment and immune infiltration analyzes, it reveals the potential mechanism by which DR influences the metabolic-immune-barrier axis of UC by regulating these three genes, thereby filling the gap in existing research concerning the association between DR and key UC genes. To further bridge the gap between bioinformatics findings and clinical practice, we developed a diagnostic nomogram based on the three identified genes. Unlike complex machine learning algorithms, this nomogram provides a visual and quantitative tool for clinicians. The exceptional diagnostic performance (AUC = 0.999) and high consistency in the calibration curve suggest that the CPT1A-ANGPTL4-CLDN1 signature could serve as a reliable, non-invasive auxiliary tool for UC risk assessment and personalized management.

The GSEA and GSVA analyzes in this study revealed that three key genes were significantly enriched in DR-associated pathways, including fatty acid metabolism and the IL2-STAT5 signaling pathway (Figure 7). This suggests that DR may influence UC progression by regulating these pathways. Mechanistically, these three genes may exhibit synergistic interactions. Carnitine palmitoyltransferase 1A (CPT1A) is localized to the mitochondrial outer membrane and serves as the key rate-limiting enzyme in fatty acid β-oxidation, responsible for catalyzing the transport of long-chain fatty acyl-CoA across the mitochondrial membrane to initiate β-oxidation, subsequently providing energy for cellular functions (Houten and Wanders, 2010; Kerner and Hoppel, 2000). Previous studies have demonstrated significantly downregulated CPT1A expression in IBD patients, which alleviates oxidative stress damage in intestinal epithelial cells and inhibits pro-inflammatory factor release by suppressing the PPARα signaling pathway (Alghamdi et al., 2024). This study, validated by RT-qPCR and database analysis, confirms downregulated CPT1A expression in ulcerative colitis (UC). This phenomenon may correlate with diet-restriction (DR)-mediated energy metabolism reprogramming: DR suppresses CPT1A expression by reducing nutrient intake, subsequently alleviating intestinal epithelial oxidative stress and inflammatory responses via the PPARα-NF-κB pathway.

Angiopoietin-like 4 (ANGPTL4), also known as fasting-inducible adipokine (FIAF) (Zuo et al., 2023). Previous studies have demonstrated its involvement in regulating intestinal epithelial cell injury (Phua et al., 2016). Li et al. demonstrated that inhibition of histone H3K27 methyltransferase EZH2 (enhancer of zeste homolog 2) markedly attenuated cell damage via epigenetic reprogramming in a lipopolysaccharide (LPS)-induced intestinal epithelial cell injury model using Caco-2 cells (Li et al., 2020). This study further validates that ANGPTL4 upregulation in UC may represent a compensatory response mediated by dietary restriction (DR): it exerts anti-inflammatory effects by inhibiting the NF-κB pathway whilst synergising with CPT1A to jointly maintain fatty acid metabolic equilibrium.

CLDN1 (Claudin-1) is a core component protein of tight junctions (TJs) (Tu et al., 2020; Cheon et al., 2025), This study identified CLDN1 as being upregulated in ulcerative colitis (UC) (validated by GSE75214/GSE73661 + RT-qPCR results), consistent with previous findings of elevated CLDN1 protein levels in the intestinal mucosa of UC patient (Devriese et al., 2017). However, this study further revealed that this phenomenon correlates with diarrhea-predominant (DR) UC—GSVA analysis revealed that butyrate metabolism pathways associated with DR were suppressed in UC, suggesting DR may cause abnormal subcellular localization of CLDN1 by reducing butyrate production. This process may interact synergistically with CPT1A-mediated. Additionally, single-cell analysis of the GSE231993 dataset provided further evidence regarding the cellular localization of these markers. CLDN1 was predominantly expressed in epithelial cells, consistent with its role in the intestinal barrier. ANGPTL4 showed enrichment in fibroblasts, while CPT1A was observed in endothelial cells. This distribution suggests that these genes may involve multiple cell types, including stromal and vascular components, in maintaining mucosal homeostasis.

Immune infiltration analysis revealed central links between our identified key genes and the UC immune microenvironment. Consistent with prior reports, neutrophil abundance was markedly elevated in UC patients (Ordás et al., 2012); neutrophil-derived elastase and reactive oxygen species (ROS) directly impair the intestinal mucosal barrier to exacerbate pathological damage. Of the 27 immune cell subsets profiled, myeloid-derived suppressor cells (MDSCs) correlated positively with activated dendritic cells (DCs), pointing to their potential role in immune tolerance dysregulation, though whether this interplay promotes T helper 17 (Th17) polarization warrants further investigation (Alghamdi et al., 2024). Conversely, cytotoxic CD56dim natural killer (NK) cells exhibited a negative correlation with T helper 2 (Th2) cells, whereby reduced NK cell levels coincided with increased Th2 cell counts (Voigt et al., 2018). Notably, IL-13 secretion by Th2 cells was significantly diminished in the intestinal mucosa of active UC patients, indicative of impaired anti-inflammatory responses, a phenotype highly consistent with goblet cell depletion driven by chronic inflammation-induced glandular atrophy. Critical to our study, most differentially abundant immune cells were positively correlated with ANGPTL4 and CLDN1 but negatively correlated with CPT1A. This distinct correlation signature not only supports a synergistic role of these three genes in orchestrating UC immune microenvironment and intestinal barrier function, but also further validates their pivotal position within the dietary restriction (DR)-mediated regulatory network underlying UC pathogenesis. CellChat analysis further suggested altered intercellular communication in the UC microenvironment. Macrophages were identified as a central signaling hub, showing increased interaction counts and strengths with fibroblasts and endothelial cells. The intensification of endothelial signaling and changes in pathways like CXCL12 suggest that the identified genes, such as ANGPTL4 in fibroblasts and CPT1A in the vasculature, may influence macrophage-related responses. This network suggests a possible link between metabolic factors and immune activity in UC.

Based on the findings of the present study, we propose an integrated, testable mechanistic hypothesis to elucidate the regulatory role of dietary restriction (DR) in ulcerative colitis (UC): DR may establish a multidimensional regulatory network encompassing metabolism, immunity, microbiota, and the barrier by synergistically modulating CPT1A, ANGPTL4, and CLDN1, thereby influencing the pathological progression of UC. Specifically, DR-mediated reduction in nutrient intake first suppresses CPT1A expression in intestinal epithelial cells, leading to impaired fatty acid β-oxidation and energy metabolism defects. This alteration may indirectly compromise CLDN1 transport and anchoring to the cell membrane by either lowering ATP levels or altering the cytoskeleton. This ultimately results in the characteristic phenotype of ‘elevated CLDN1 protein levels but compromised barrier function’ (verifiable by CPT1A knockdown in intestinal epithelial cells followed by CLDN1 subcellular localization analysis); Concurrently, CPT1A-mediated inhibition of fatty acid oxidation may trigger compensatory upregulation of ANGPTL4. As a pivotal crossroads between inflammation and metabolism, ANGPTL4 mitigates intestinal inflammation by suppressing the NF-κB pathway whilst simultaneously participating in cellular energy source reprogramming to counteract metabolic stress induced by CPT1A downregulation (verifiable by concurrently regulating both gene expressions and observing changes in inflammatory levels and metabolic phenotypes in UC models). Importantly, our single-cell analysis suggests that this process is integrated into a broader multicellular network beyond the epithelium. While epithelial CLDN1 localization is impaired, the compensatory upregulation of ANGPTL4 in fibroblasts and the altered communication between endothelial cells and macrophages (e.g., via ACKR1 signaling) collectively shape the inflammatory landscape of the colonic mucosa. Moreover, the dysregulation of CLDN1 localization is closely associated with DR-related gut microbial metabolic disorders—DR may impair microbial metabolic efficiency of dietary fiber, leading to reduced butyrate production. While diminished CPT1A activity may further impair epithelial cells’ butyrate utilization capacity. Together, these form the core components of the ‘dietary fiber-microbial metabolism-host barrier function’ regulatory axis (verifiable by supplementing butyrate and assessing changes in the expression, localization, and barrier function of the three genes). This integrated hypothesis tightly links DR with core pathological features of UC (metabolic reprogramming, inflammatory imbalance, barrier damage, microbial dysregulation) through key genes, providing a clear experimental direction for further elucidating DR’s anti-UC mechanism.

This study has several inherent limitations that should be acknowledged. First, the bioinformatics analyzes relied on public datasets, which may be subject to sample heterogeneity; additionally, validation using multi-center clinical samples is lacking. Second, only mRNA expression levels of the identified genes were verified by RT-qPCR. The limited sample size (n = 5 per group) precluded robust stratified analyses. Furthermore, protein-level validation (e.g., IHC to detect the subcellular localization of CLDN1) was not performed. Moreover, functional experiments to confirm gene-gene interactions and the regulatory role of DR remain absent. Third, the DR-related gene set (DRRGs) was screened based on existing studies, potentially resulting in the omission of critical genes. Furthermore, the risk of overfitting remains a potential limitation despite the high AUC values. The nomogram was developed using specific public datasets, which may contain inherent biases or noise. Although we employed cross-calibration and multiple algorithms to mitigate this risk, the generalizability of the CPT1A-ANGPTL4-CLDN1 signature requires further validation in larger, multi-center external clinical cohorts. To address these limitations and further advance this field, future studies should focus on the following aspects: (1) Expand the clinical sample size, and combine with DR intervention history to verify the correlation between the expression of the three key genes (CPT1A, ANGPTL4, CLDN1) and DR; (2) Utilize DR animal models and cell experiments to validate the aforementioned gene interaction hypotheses and pathway regulatory mechanisms; (3) Supplement protein-level validation (e.g., IHC, Western blot); to clarify the relationship between the subcellular localization of CLDN1 and DR.

In conclusion, this study focused on screening the key genes (ANGPTL4, CLDN1, and CPT1A) in UC and revealing their important roles in disease progression. However, this study also has some limitations, such as limited sample size and research methods, which may lead to some differences between the results of the study and those of previous reports. We will continue to focus on the role of these mechanisms in UC and plan to further expand the sample size, optimize research methods, and explore more deeply the specific roles of these key genes in the occurrence and progression of the disease in future studies, thereby providing new insights and strategies for the treatment of UC.

## Data Availability

The datasets presented in this study can be found in online repositories. The names of the repository/repositories and accession number(s) can be found in the article/Supplementary Material.

## Glossary

UC: Ulcerative colitis
DR: Dietary restriction
DRRGs: DR-related genes
DEGs: Differentially expressed transcripts
DCA: Decision curve analysis
AUROC: Area under the receiver operating characteristic
GSEA: Gene Set Enrichment Analysis
GSVA: Gene Set Variation Analysis
IBD: Inflammatory bowel disease
5-ASA: 5-aminosalicylic acid
GEO: Gene Expression Omnibus
GO: Gene Ontology
FC: Fold Change
KEGG: Kyoto Encyclopedia of Genes and Genomes
CC: Cellular component
MF: Molecular function
BP: Biological process
STRING: Search Tool for the Retrieval of Interacting Genes/Proteins
PPI: Protein-protein interaction
BC: Betweenness centrality
EPC: Edge Percolated Component
MCC: Maximal Clique Centrality
LASSO: Least absolute shrinkage and selection operator
SVM-RFE: Support vector machine recursive feature elimination
ROC: Receiver operating characteristic
AUC: Area under the receiver operating characteristic curve
DNN: Deep neural network
MSigDB: Molecular Signatures Database
NES: Normalized Enrichment Score
DICs: Differential immune cells
TFs: Transcription factors
RT-qPCR: Quantitative reverse transcription polymerase chain reaction
GAPDH: Gene glyceraldehyde-3-phosphate dehydrogenase
ANGPTL4: Angiopoietin-like protein 4
CLDN1: Claudin-1
CPT1A: Carnitine palmitoyltransferase 1A
MDSCs: Myeloid-derived suppressor cells
FDA: Food and Drug Administration
FIAF: Fasting-inducible adipokine
DSS: Dextran sulfate sodium
NF-κB: Nuclear factor kappa B
TJs: Tight junctions
LPS: Lipopolysaccharide
