# Supplementary material for: Revealing key genes and molecular mechanisms associated with dietary restriction in ulcerative colitis
Source: Front Mol Biosci. 2026 Mar 25;13:1786138. doi: 10.3389/fmolb.2026.1786138 (PMC13056633; doi:10.3389/fmolb.2026.1786138)
Supplement: Supplementary file 1 [file Supplementaryfile1.zip › Supplementary Tables and Figures/Supplementary Table 20.docx]

| **primer** | **sequence** | |
| --- | --- | --- |
| ANGPTL4 F | CCTCTCCGTACCCTTCTCCA | |
| ANGPTL4 R | AAACCACCAGCCTCCAGAGA | |
| CLDN1 F | CTGGGAGGTGCCCTACTTTG |  |
| CLDN1 R | ACACGTAGTCTTTCCCGCTG |  |
| CPT1A F | GCAGCGTTCTTTGTGACGTT |  |
| CPT1A R | AGGAGTGTTCAGCGTTGAGG |  |
| internal reference-GAPDH F | ATGGGCAGCCGTTAGGAAAG |  |
| internal reference-GAPDH R | AGGAAAAGCATCACCCGGAG |  |
