# Supplementary figures and images for: Revealing key genes and molecular mechanisms associated with dietary restriction in ulcerative colitis
Source: Front Mol Biosci. 2026 Mar 25;13:1786138. doi: 10.3389/fmolb.2026.1786138 (PMC13056633; doi:10.3389/fmolb.2026.1786138)

GenDR

DRRGs

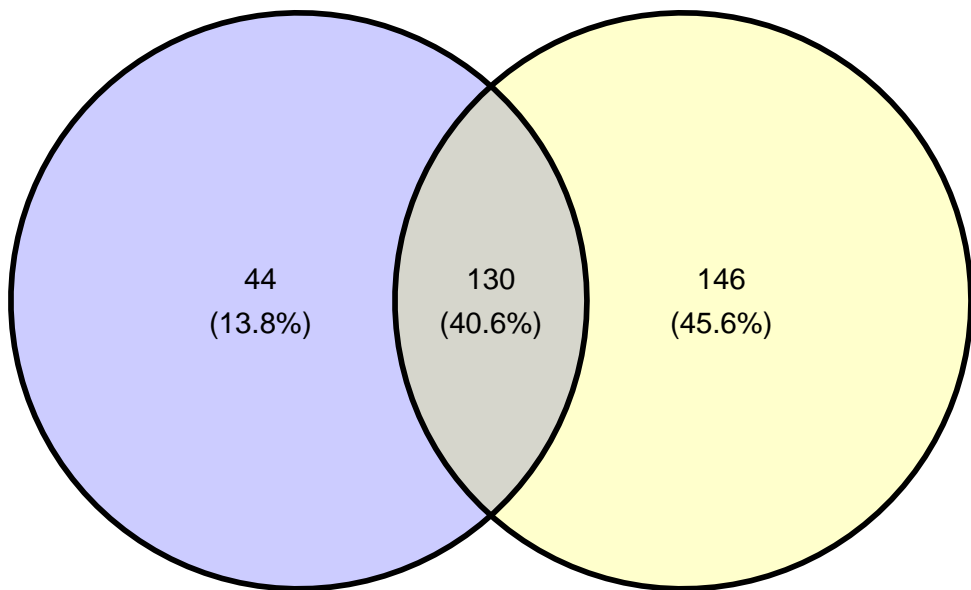

Supplement: Supplementary file 1 [file Supplementaryfile1.zip › Supplementary Tables and Figures/Supplementary Figure 1.pdf]

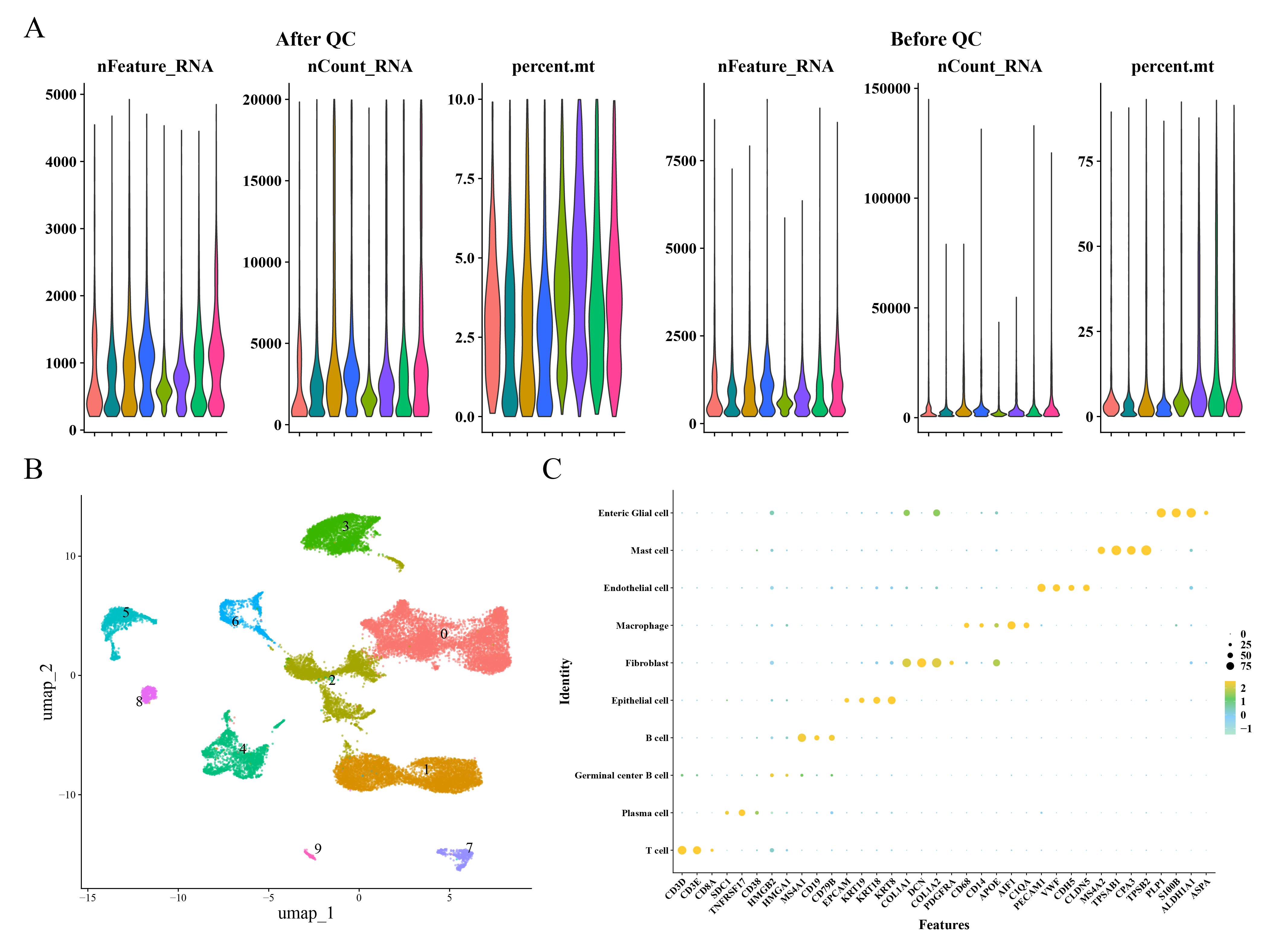

Supplement: Supplementary file 1 [file Supplementaryfile1.zip › Supplementary Tables and Figures/Supplementary Figure 2.tif]
