# Supplementary material for: Revealing key genes and molecular mechanisms associated with dietary restriction in ulcerative colitis
Source: Front Mol Biosci. 2026 Mar 25;13:1786138. doi: 10.3389/fmolb.2026.1786138 (PMC13056633; doi:10.3389/fmolb.2026.1786138)
Supplement: Supplementary file 1 [file Supplementaryfile1.zip › Supplementary Tables and Figures/Supplementary_Material.doc]

Supplementary Material

# 1 Supplementary Data

Supplementary tables and figures have been uploaded separately on submission.

# 2 Supplementary Tables

**Supplementary Table 1.** List of DRRGs.

**Supplementary Table 2.** List of DEGs.

**Supplementary Table 3.** List of candidate genes.

**Supplementary Table 4.** GO enrichment.

**Supplementary Table 5.** KEGG enrichment.

**Supplementary Table 6.** List of genes from the BC.

**Supplementary Table 7.** List of genes from the MCC.

**Supplementary Table 8.** List of genes from the EPC.

**Supplementary Table 9.** List of genes from the RC.

**Supplementary Table 10.** GSEA enrichment analysis focusing on CPT1A.

**Supplementary Table 11.** GSEA enrichment analysis focusing on ANGPTL4.

**Supplementary Table 12.** GSEA enrichment analysis focusing on CLDN1.

**Supplementary Table 13.** GSVA enrichment analysis.

**Supplementary Table 14.** Analysis of correlation.

**Supplementary Table 15.** 39 common miRNAs.

**Supplementary Table 16.** List of TFs.

**Supplementary Table 17.** Predicted drug list for ANGPTL4.

**Supplementary Table 18.** Predicted drug list for CLDN1.

**Supplementary Table 19.** Predicted drug list for CPT1A.

**Supplementary Table 20.** Oligonucleotide primer sequences.

**Supplementary Table 21.** List of genes consistently overexpressed (a,) and underexpressed (b,) with CR.

**Supplementary Table 22.** Marker genes list.

**Supplementary Table 23.** The clinical characteristics of the enrolled participants.

# 3 Supplementary Figures

**Supplementary Figure 1.** The Venn diagram.

**Supplementary Figure 2.** Quality control and cell type annotation of the scRNA-seq dataset (GSE231993). (A) Violin plots illustrating the quality control (QC) metrics before and after filtering. (B) UMAP visualization showing the initial 10 clusters (numbered 0–9) based on unsupervised clustering before biological annotation. (C) Dot plot of canonical marker genes used to identify and annotate the 10 cell lineages.
